# Chromosome numbers and DNA content in some species of *Mecardonia* (Gratiolae, Plantaginaceae)

**DOI:** 10.3897/CompCytogen.v10i4.10362

**Published:** 2016-12-14

**Authors:** María M. Sosa, María B. Angulo, Julián A. Greppi, Verónica Bugallo

**Affiliations:** 1 Instituto de Botánica del Nordeste (UNNE-CONICET). Sargento Cabral 2131. Facultad de Ciencias Exactas, Naturales y Agrimensura (UNNE). Av. Libertad 5460. 3400. Corrientes, Argentina; 2 Instituto de Floricultura, Instituto Nacional de Tecnología Agropecuaria, Hurlingham 1686, Buenos Aires, Argentina

**Keywords:** Gratiolae, chromosome number, DNA content, flow cytometry, polyploidy

## Abstract

Cytogenetic characterization and determination of DNA content by flow cytometry of five species of *Mecardonia* Ruiz et Pavon, 1798 (Gratiolae, Plantaginaceae) was performed. This is the first study of nuclear DNA content carried out in the genus. Mitotic analysis revealed a base chromosome number x = 11 for all entities and different ploidy levels, ranging from diploid (2n = 2x = 22) to hexaploid (2n = 6x = 66). The results include the ﬁrst report of the chromosome numbers for *Mecardonia flagellaris* (Chamisso & Schlechtendal, 1827) (2n = 22), *Mecardonia grandiflora* (Bentham) Pennell, 1946 (2n = 22), *Mecardonia kamogawae* Greppi & Hagiwara, 2011 (2n = 66), and *Mecardonia* sp. (2n = 44). The three ploidy levels here reported suggest that polyploidy is common in *Mecardonia* and appear to be an important factor in the evolution of this genus. The 2C- and 1Cx-values were also estimated in all the species. The 2C-values ranged from 1.91 to 5.29 pg. The 1Cx-values ranged from 0.88 to 1.03 pg. The general tendency indicated a decrease in the 1Cx-value with increasing ploidy level. The signiﬁcance of the results is discussed in relation to taxonomy of the genus.

## Introduction

*Mecardonia* Ruiz & Pavon, 1798 belongs to the tribe Gratiolae (Plantaginaceae) and is distributed across the America, reaching its southernmost distribution in Argentina. The species are erect or creeping herbs, annual or perennial, much branched, mostly glabrous, sometimes blackening on drying, gland dotted, and yellow and white flowers (D’Arcy 1979). Since the description of the genus (Ruiz and Pavon 1794), there have been a few problems in establishing its generic and infrageneric circumscription. Rossow (1987) considered only 10 species, which had been previoulsy placed in *Bacopa* Aublet, 1775 under subgenus Mecardonia (Descole and Borsini 1954). More recently, Souza (1997) and Souza and Giuletti (2009) carried out some taxonomic modifications to Rossow’s clasiffication. The demarcation of the genus is variable depending on the author consulted. Following Rossow’s classiﬁcation, the genus includes five species growing in Argentina: *Mecardonia flagellaris* (Chamisso & Schlechtendal, 1827), *Mecardonia grandiflora* (Bentham) Pennell, 1946, *Mecardonia procumbens* Small, 1903, *Mecardonia serpylloides* (Chamisso & Schlechtendal, 1891) and *Mecardonia tenella* (Chamisso & Schlechtendal, 1891). Recently, *Mecardonia kamogawae* Greppi & Hagiwara, 2011 was described by as an endemic species of Corrientes Province (Argentina).

*Mecardonia* has ornamental value because some cultivars developed from native species from Northern Argentina were recently introduced in the trade of ornamental plants (Greppi 2012). Therefore, researches on genetic improvement are carried out in this genus.

Cytological and cytogenetic studies have proved useful data for taxonomic and evolutionary analyses, which are widely used in processes of conventional or biotechnological genetic improvement (Poggio et al. 2004). Characters such as chromosome number, morphology, and meiotic behavior, as well as nuclear DNA content, have been used as taxonomic markers helping to circumscribe taxa and infer their relationships (Kron et al. 2007, Guerra 2008, Loureiro et al. 2010, Castro et al. 2012). At present, only two species of *Mecardonia* have been evaluated cytologically. Lewis et al. (1962) reported 2n = 42±2 for a Northamerican species *Mecardonia acuminata* (Walter, 1891) Small, 1903. Kaul (1969) determined 2n = 2x = 22 for *Mecardonia procumbens* Small, 1903 (as *Mecardonia dianthera* (Swartz 1900, Pennell 1946). Therefore, to increase the knowledge of *Mecardonia*, other species were cytologically analyzed in this study.

Nuclear DNA content, understood as genome size, is very variable across angiosperm, and has been revealed as an important character in biodiversity. In *Mecardonia* species there are no reported measurements of DNA content, but genome size variation has been explored in some genera of Plantaginaceae. DNA C-values are currently available for 204 species belonging to 18 genera of this family, including *Callitriche* Linnaeus, 1753, *Penstemon* Schmidel, 1762, *Plantago* Linnaeus, 1753 and *Veronica* Linnaeus, 1753 with a range of variation of 0.32–4,.63 pg (Albach and Greilhuber 2004, Broderick et al. 2011, Bennett and Leitch 2012, Wong and Murray 2012, Prančl et al. 2014, Meudt et al. 2015). Herein we used ﬂow cytometry to estimate the nuclear DNA content in five species of *Mecardonia* for the ﬁrst time. Additionally, we report original chromosome numbers of some of them. The results are discussed in relation to the taxonomy and evolution of the genus.

## Materials and methods

We examined six populations from five species of *Mecardonia* collected in Argentina. Information about the studied material and the voucher specimens is provided in Table 1. Vouchers are deposited at the herbarium BAB of the Instituto Nacional de Tecnología Agropecuaria (INTA), Buenos Aires, Argentina.

**Table 1.** *Mecardonia* species analyzed in this study, with their respective chromosome numbers (2n), locations, and voucher specimens.

	Species	2n	Location, voucher specimen
*	*Mecardonia flagellaris* (Cham. & Schldlt.) Rossow	2n = 2x = 22	Argentina. Entre Ríos, Dept. Federación, in front of complejo turístico Irupé. Greppi et al. 1411 (BAB).
		2n = 2x = 22	Argentina. Entre Ríos, Dept. Federación, complejo turístico Irupé Greppi et al. 1190 (BAB).
*	*Mecardonia grandiflora* (Benth.) Pennell	2n = 2x = 22	Argentina. Misiones, Dept. Guaraní, Ayo. Pepirí Miní o Yabotí. Greppi et al. 1189 (BAB).
*	*Mecardonia kamogawae* Greppi & Hagiwara	2n = 6x = 66	Argentina. Corrientes, Dept. Paso de los Libres, Paso de los Libres to national route 14, Greppi et al. 1081 (BAB).
	*Mecardonia procumbens* (Mill.) Small	2n = 2x = 22	Argentina. Córdoba. Dept. Unión, Monte Leña, national route 9, km 491, Greppi 681(BAB).
*	*Mecardonia* sp. n.	2n = 4x = 44	Argentina. Corrientes. Dept. Empedrado. Greppi and Hagiwara 1410 (BAB)

* First chromosome count.

### Mitosis analysis

Mitotic chromosome preparations were made from root meristems obtained from rooted stems. The roots were pretreated for about 4 h in 0.002 M 8-hydroxyquinoline solution at room temperature, ﬁxed in 5:1 absolute alcohol/lactic acid, and then stained using Feulgen’s technique. Permanent microscope slides were prepared by mounting in Euparal. In all samples at least 20 counts of 7–10 individuals were made to verify the observations.

Permanent microscope slides were examined and photographed using Zeiss Axioplan microscope Carl Zeiss with digital camera Canon Power Shot A 640.

### Nuclear DNA measurements

DNA content (in picograms) was estimated by ﬂow cytometry using fresh young leaves. The measurements were calculated from three replicates per individuals. In total we analyzed three individuals per species. The leaves of *Zea mays* Linnaeus, 1753 cv. ‘CE-777’ (2C = 5.43 pg., Doležel et al. 1998) were used as internal standard for almost all entities. While, *Hordeum vulgare* Linnaeus, 1753 cv. ‘New Golden’ (2C = 10.4 pg., Bennett and Leitch 2005) was used as the standard of hexaploid species. The selection of these internal standards was made since they are the common standards used in the laboratory where the ﬂow cytometer is situated (Instituto de Floricultura, INTA Castelar, Buenos Aires, Argentina). To release nuclei from the cells, 0.5 cm^2^ of leaf tissue of *Mecardonia* was chopped together with 0.5 cm^2^ of leaf tissue of the internal standard in 0.5 ml buffer (High resolution DNA kit, Partec GmbH, Münster, Germany). Subsequently, 5 U ml ^-1^ of RNAse were added and incubated for 2–5 min at room temperature. The suspension was ﬁltered through a 30 µm nylon mesh. After this period, 1.5 ml of staining solution containing 1µg µl^-1^ propidium iodide was added. Within 1 h of staining, measurements were performed with a CyFlow Ploidy Analyzer, Partec cytometer (green laser 532 nm, 30 mW). About 10,000 nuclei were measured for each sample.

The absolute value of DNA content (2C) of each sample was calculated by the formula: (X peak of sample × G1 DNA content (2C) of the standard)/X G1 peak of the standard (Doležel and Bartos 2005).

The monoploid genome size (1Cx) was calculated dividing the 2C-value by the ploidy level (Greilhuber et al. 2005).

### Data analysis

The mean, standard deviation and the coefﬁcient of variation of 2C-value were calculated for each species from three different individuals. Differences in 1Cx-value between species were tested by one-way analysis of variance (ANOVA) at a signiﬁcance level of 5% (a = 0.05). The Tukey 5% post hoc test was used to test differences between each pair of species.

Pearson correlation coefﬁcient was calculated to test whether the 2C–and 1Cx-values were related to chromosome number. Scatter plot was performed to evaluate the relationship between the 1Cx-values and the chromosome numbers (2n) of species. All statistical analyses were performed using the InfoStat software version 2013 (Di Rienzo et al. 2013).

## Results

**Figure 1.**
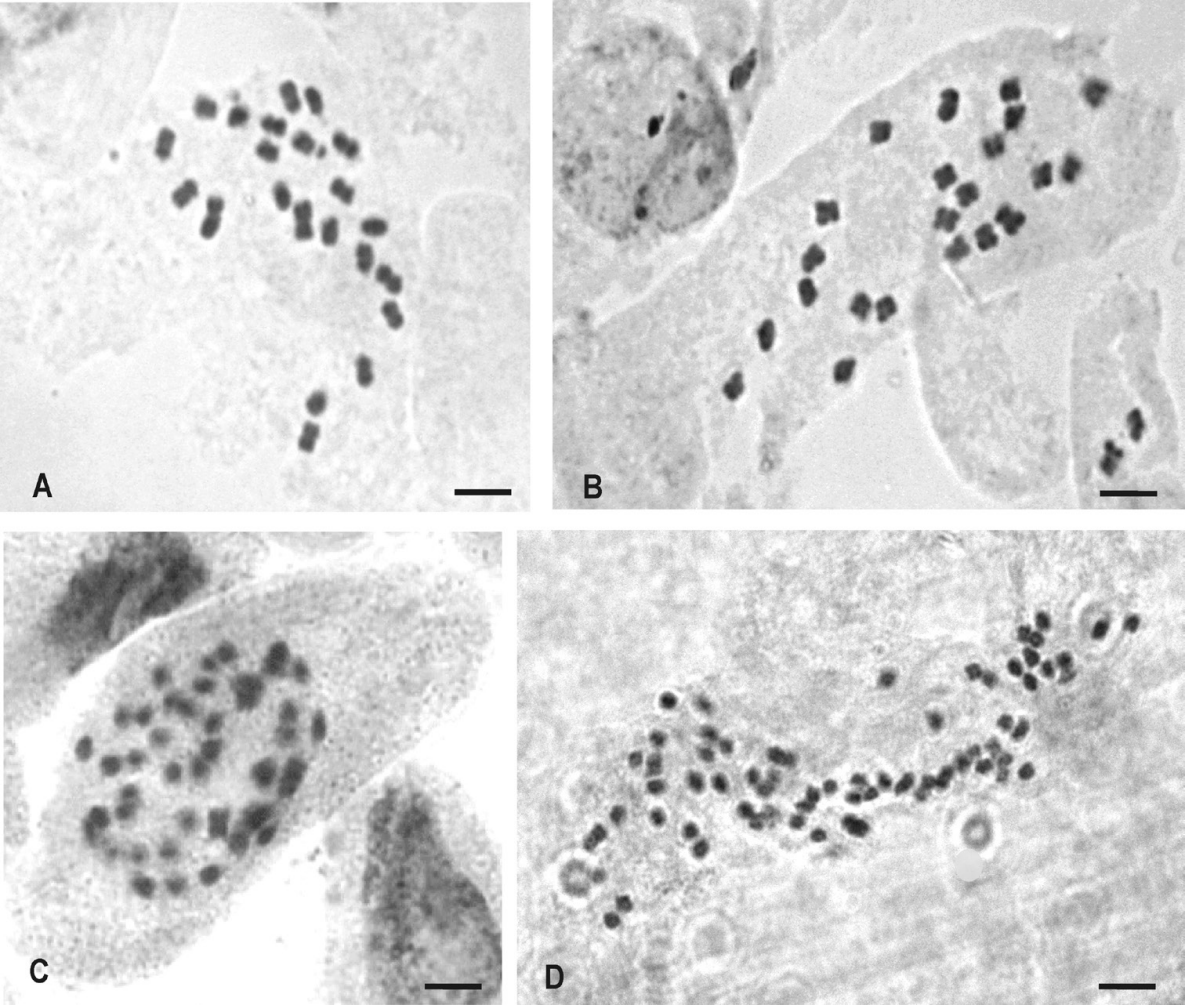
Somatic chromosomes of *Mecardonia* species. **A** *Mecardonia flagellaris*: 2n = 2x = 22 **B** *Mecardonia grandiflora*: 2n = 2x = 22 **C** *Mecardonia* sp. n.: 2n = 4x = 44 **D** *Mecardonia kamogawae*: 2n = 6x = 66. Bar = 5 μm.

The chromosome numbers of six populations belonging to five species of *Mecardonia* were determined. The analyzed species and their chromosome numbers are given in Table 1. Four species were analyzed for the first time. The chromosome number observed in the remaining taxon is in agreement with previous studies. All species analysed shared the same base chromosome number (x = 11), and chromosome numbers ranged between 2n = 22 to 2n = 66. Of these, only three species were diploids: *Mecardonia flagellaris* (Fig. 1 A), *Mecardonia grandiflora* (Fig. 1 B) and *Mecardonia procumbens*. The remaining species were polyploids, *Mecardonia* sp. (Fig. 1 C) was tetraploid with 2n = 44 and *Mecardonia kamogawae* was hexaploid with 2n = 66 (Fig. 1 D).

### Genomic DNA content

The DNA amounts determined for five species of *Mecardonia* are shown in Table 2. The flow cytometric measurements of all species and the standards resulted in well defined and sharp peaks. In all cases, the coefﬁcients of variation were lower than 5% (Table 2), supporting the reliability of the ﬂow cytometric assessments.

**Table 2.** Chromosome number (2n), ploidy level, 2C-value (pg), CV (coefficient of variation), 1Cx-value (pg) of the *Mecardonia* species analyzed.

**Species**	**Chromosome number (2n)**	**Ploidy level**	**2C (pg)**	**CV**	**1Cx (pg)**
*Mecardonia flagellaris*	22	2x	2.06 ± 0.16	0.029	1.03^d^
*Mecardonia grandiflora*	22	2x	2.05 ± 0.08	0.010	1.02^d^
*Mecardonia procumbens*	22	2x	1.91 ± 0.06	0.012	0.95^c^
*Mecardonia* sp. n.	44	4x	3.71 ± 0.05	0.053	0.92^b^
*Mecardonia kamogawae*	66	6x	5.29 ± 0.10	0.061	0.88^a^
ANOVA					(F=357.52; P= <0.0001)

For ANOVA results, different lower-case letters indicate significant differences among species for mean values of each parameter at 5% level using Tukey’s test.

The 2C-values of the species here analyzed varied from 1.91 pg in *Mecardonia procumbens* (2x) to 5.29 pg in *Mecardonia kamogawae* (6x). The 2C-values were strongly and signiﬁcantly correlated with chromosome number (r= 0.99; P= < 0.0001).

**Figure 2.**
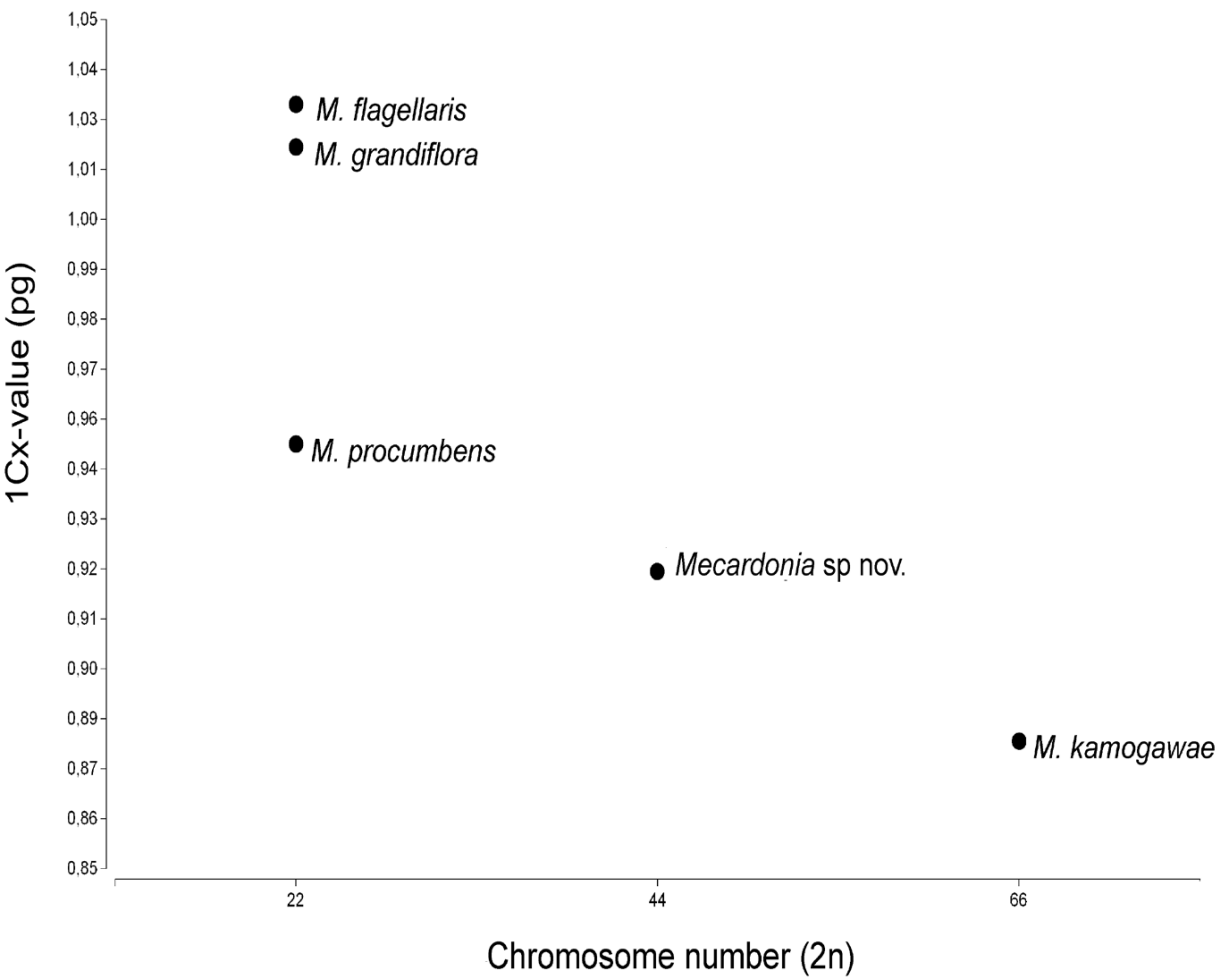
Scatter plot between 1Cx-value and chromosome number (2n).

The 1Cx-values, which indicated the DNA content per genome, ranging from 1Cx= 0.88 pg in *Mecardonia kamogawae* to 1Cx= 1.03 pg in *Mecardonia flagellaris* (Table 2). The ANOVA showed signiﬁcant differences for 1Cx-values (F= 357.52; P= <0.0001) between the species. The correlation between values of 1Cx and chromosome number was negative and signiﬁcant (r= -0.86; P= <0.0001, Fig. 2).

## Discussion

The chromosome number 2n = 22 found in *Mecardonia procumbens*, is consistent with the chromosome counts recorded in a previous cytological study (Kaul 1969). Sinha (1987) reported B chromosomes for this taxon; however, the populations here analyzed did not show these accessory chromosomes. Chromosome counts for *Mecardonia flagellaris* (2n = 22), *Mecardonia grandiflora* (2n = 22), *Mecardonia kamogawae* (2n = 66) and *Mecardonia* sp. (2n = 44) are described here for the first time. Our results showed diploid and polyploid species for the genus. Polyploidization has long been recognized as an important process in plant evolution (Otto and Whitton 2000, Soltis et al. 2004). In Plantaginaceae, polyploidy is a common phenomenon occuring in many genera, such as *Antirrhinum* Linnaeus, 1753, *Chaenorhinum* (DC.) Reichenbach, 1828, *Cymbalaria* Hill, 1756, *Chelone* Linnaeus, 1753, *Digitalis* Linnaeus, 1753, *Linaria* Miller, 1754, *Plantago*, *Nuttallanthus* D.A. Sutton, 1988, *Stemodia* Linnaeus, 1759, *Veronica* (Hair 1966, Subramanian and Pondmudi 1987, Sosa and Seijo 2002, Sosa et al. 2009, 2011, Wolfe et al. 2002, Vargas et al. 2004, Murray et al. 2010, Castro et al. 2012, Wong and Murray 2012, Ranjbar and Nouri 2015). Our results evidenced the presence of multiple cytotypes in *Mecardonia*, hence suggesting polyploidy as a key driver of the evolution of the genus. The present analysis, in addition to previous chromosome number reports, revealed that the genus have exclusively the basic chromosome number of x = 11.

The interest on the study of genome size increased in the last decade. These studies focused on the use of genome size as a taxonomic marker (Castro et al. 2012, Angulo and Dematteis 2013, Galdeano et al. 2016) and on finding correlations between ecological and environmental variables and this character (Chalup et al. 2014, Vega and Dematteis 2015). However, there are still many families and genera being neglected, including *Mecardonia*, for which the present study is the first analysis of genome size for the genus. The estimates 2C- and 1C-values calculated for the species in this study are within ranges of variation found in Angiosperms and Plantaginaceae (Leitch and Bennett 2004, Meudt et al. 2015). Based on the available genome size data, *Mecardonia* falls into the categories “very small” genomes (2C = <2.8 pg) to “small” genomes (2C = <7.0 pg) according to values reported by Leitch et al. (1998) and Soltis et al. (2004).

The 2C-values of *Mecardonia* species revealed a positive and significant correlation with chromosome number (r = 0.99, P = < 0.0001). Therefore, in the genus there is a trend for increasing 2C–value with increasing ploidy level. On the other hand, the variation of 1Cx-values is negative and significantly (r = -0.86; P = <0.0001) correlated with chromosome number. Consequently, the values of 1Cx of the species decrease in inverse proportional to the ploidy level. Our data reflect that both polyploids (tetraploid 1Cx = 0.92 pg and hexaploid 1Cx = 0.88 pg) have lesser values of monoploid genome size than diploid species (mean of Cx = 1.00 pg). Many polyploid angiosperms undergo *genome downsizing* and so have smaller average genome sizes than their diploid relatives (Leitch and Bennett 2004, Leitch et al. 2008) and *Mecardonia* seems not to be an exception. Several studies have indicated that during polyploidization different balancing processes at genomic level occur which may promote variation in nuclear DNA content. These changes point towards a possible need for harmonization of genome and removal of some unnecessary genomic redundancies (Petrov 2001; Bennetzen et al. 2005, Pellicer et al. 2010). Mechanisms leading to a decrease in genome size in polyploids may include non-random elimination of chromosome- and genome-speciﬁc sequences (Ozkan et al. 2003, Shaked et al. 2001), illegitimate crossing over (Devos et al. 2002) or unbalanced deletion–insertion rates (Petrov 2001, 2002). Counterbalancing mechanisms are probably also involved to reduce the genetic and structural instabilities that accompany DNA loss (Pellicer et al. 2010).

Recently, Meudt et al. (2015) established a relationship between the genome downsizing with diversiﬁcation in polyploid lineages of *Veronica* (Plantaginaceae), but they do not know how general this pattern might be or what causes it. Several hypotheses have been proposed to explain this relationship. Kraaijeveld (2010) suggested that organisms with small genomes have more stably inherited mutations, or a nucleotypic effect, in which organisms with small genomes and shorter genes have a general advantage as a result of faster replication and transcription. The genome size changes in *Mecardonia* are probably related with the diversification of the species. Further studies comparing this genus with the closest extant relative to determine what aspect of genome downsizing facilitate diversification are needed.

## Taxonomic implications

The genus *Mecardonia* is currently under revision and some closely related species with intermediate morphological characteristics were found. It has been well documented in many plants that chromosome numbers and genome size can be used as extra taxonomic characters for discriminating between closely related taxa, helping to clarify the taxonomy of some species in problematic genus (Guerra 2008, Castro et al. 2012, Sosa and Dematteis 2014). For example, *Mercardonia* sp. is closely related to *Mecardonia flagellaris*. A detailed morphological analysis along with chromosome number here reported showed that it should be considered as different species. *Mecardonia* sp. is tetraploid with 2n = 44, while *Mecardonia flagellaris* is diploid with 2n = 22. Thus, both species differ in chromosome number and morphological features, such as aspect of plant, leaf shape, and trichome types of corolla. In addition, the new species has more restricted distribution to North of Argentina. However, *Mecardonia flagellaris* is expanding from Mato Grosso do Sul (Brazil) to Chubut (Argentina), arrived to Chile, Paraguay and Uruguay.

Another case is *Mecardonia kamogawae* that is morphologically related to *Mecardonia procumbens* from which it differs in the life-form, root types, leaf texture, and size of bracteoles and pedicels. Regarding chromosome number, *Mecardonia kamogawae* is hexaploid with 2n = 66, while *Mecardonia procumbens* is diploid with 2n = 22. Therefore, both species can be distinguished by morphological features, as well as by the chromosome number.

*Mecardonia procumbens* and *Mecardonia flagellaris* were diploids with 2n = 22. Although the chromosome number does not distinguish both species, differences in 2C-values were observed. *Mecardonia flagellaris* had higher value (2C = 2.06 pg.) than *Mecardonia procumbens* (2C = 1.91 pg.). D’Arcy (1979) and Souza (1997) placed *Mecardonia flagellaris* under the synonymy of *Mecardonia procumbens* by having similar morphological characteristics. We considered, however, that both species are morphologically distinct. *Mecardonia procumbens* differs by aspect of the plant, shape and length of leaf, and calyx pieces. Also, *Mecardonia procumbens* has a wider distribution as it extends from the South of the United States of America to the Argentine Northwest. However, *Mecardonia flagellaris* grow in Paraguay, South of Brasil, Uruguay and Northeast of Argentina.

## Conclusion

The results of this study suggest that chromosome number is useful in distinguishing species of *Mecardonia*. The different ploidy levels of the taxa showed the importance of polyploidy in the evolution of the genus. The results here obtained combined with those reported previously conﬁrm that the *Mecardonia* genus has basic number x = 11.

Regarding to the variation of genome size, decreases in DNA content have occurred during the evolution of genome size in the *Mecardonia* species.

Our results showed that differences in morphological features along with chromosome numbers and DNA content values support Rossow’s criterion (1987).
